# Supplementary figures and images for: Glypican-1 drives unconventional secretion of fibroblast growth factor 2
Source: eLife. 2022 Mar 29;11:e75545. doi: 10.7554/eLife.75545 (PMC8986318; doi:10.7554/eLife.75545)

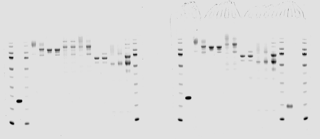

Supplement: Figure 6—figure supplement 1—source data 1. — Figure 6. [file elife-75545-fig6-figsupp1-data1.zip › Figure 6 - Figure Supplement 1 - Source data 1.tiff]

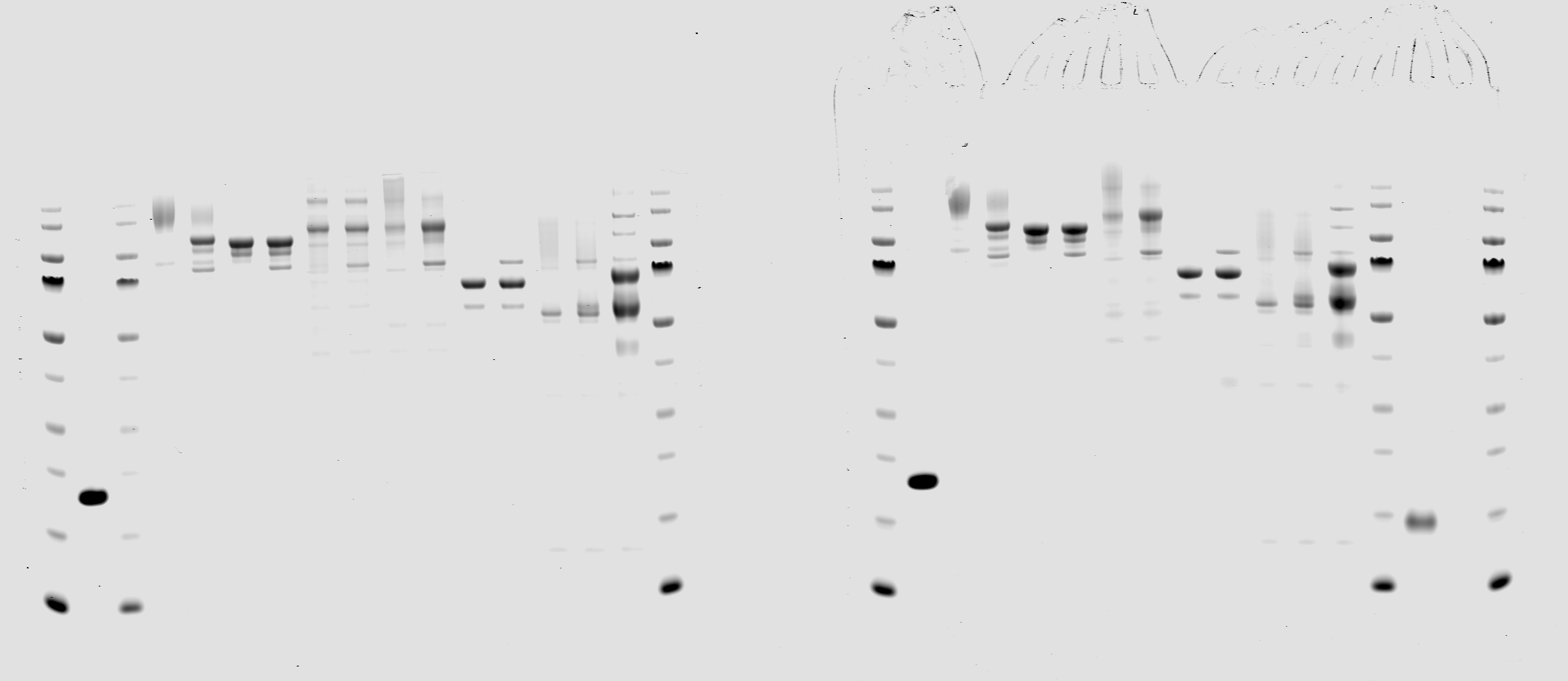

Supplement: Source data 1. [file elife-75545-data1.zip › 2022-02-25 source data 3/Figure 6 - Figure Supplement 1 - Source data 1.tif]

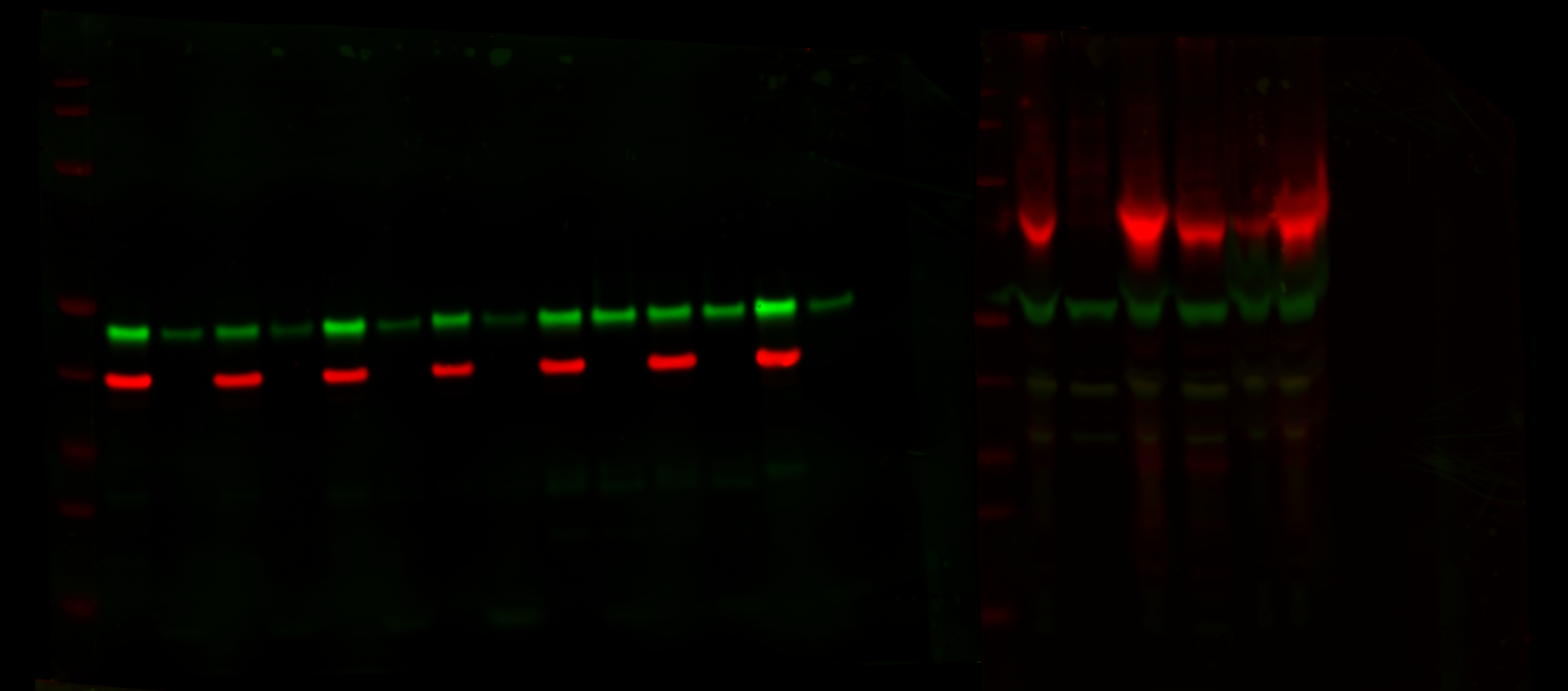

Supplement: Source data 1. [file elife-75545-data1.zip › 2022-02-25 source data 3/Figure 4 - Source data/Figure 4 - Source data 3.tif]

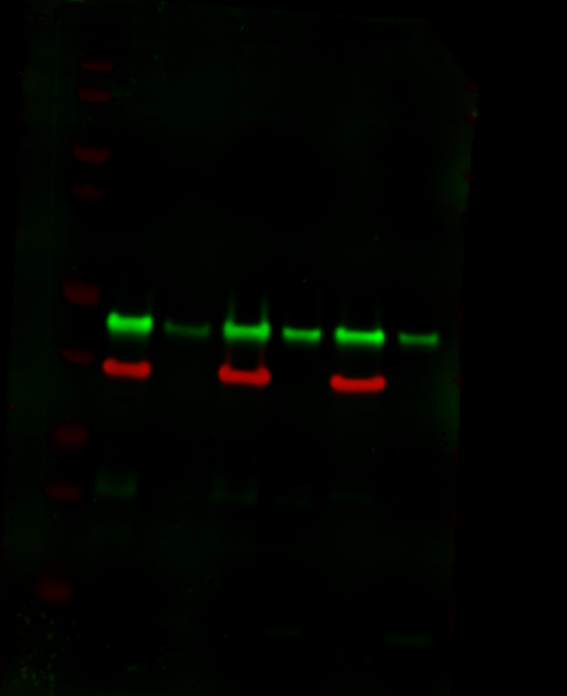

Supplement: Source data 1. [file elife-75545-data1.zip › 2022-02-25 source data 3/Figure 4 - Source data/Figure 4 - Source data 2.tif]

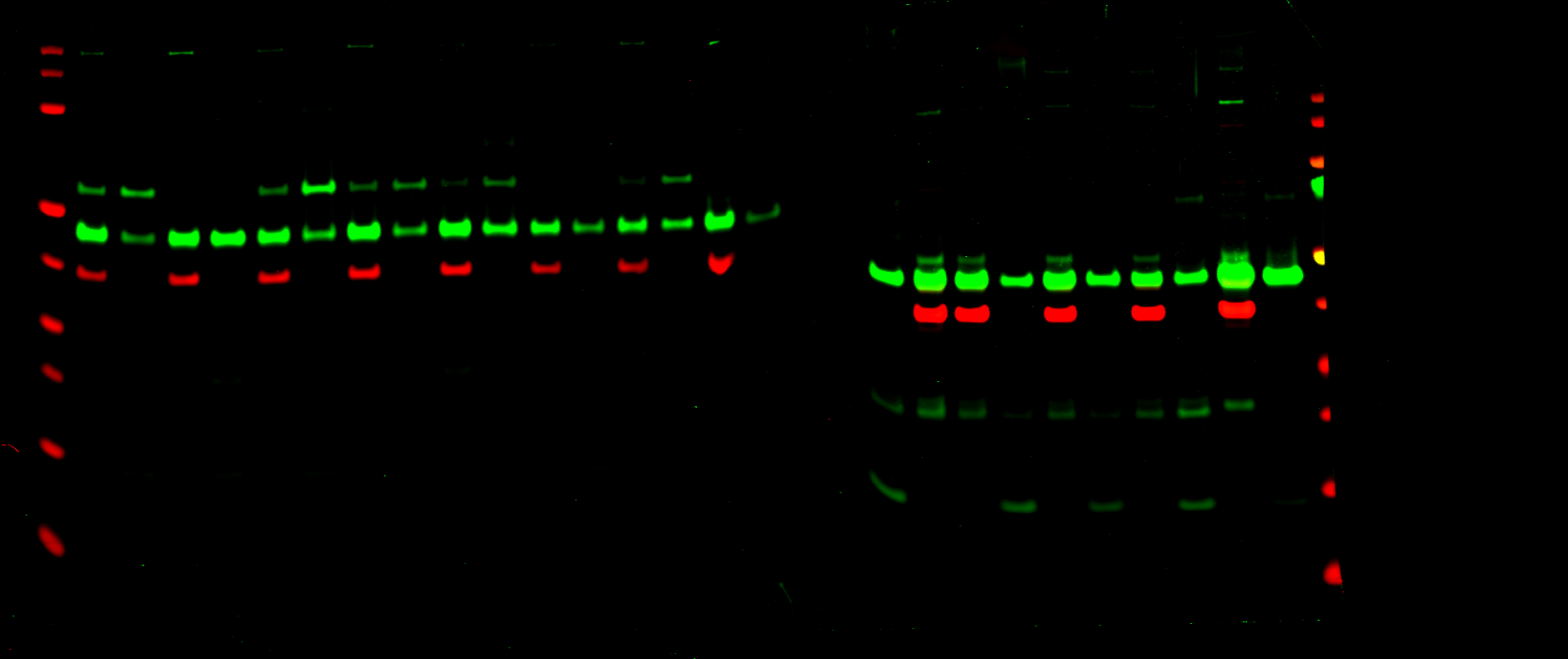

Supplement: Source data 1. [file elife-75545-data1.zip › 2022-02-25 source data 3/Figure 4 - Source data/Figure 4 - Source data 1.tif]

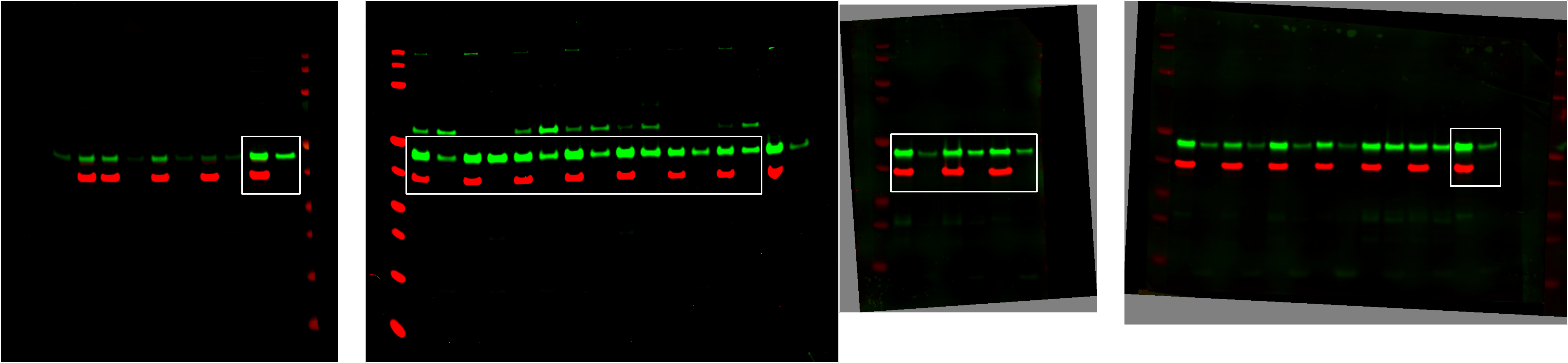

Supplement: Source data 1. [file elife-75545-data1.zip › 2022-02-25 source data 3/Figure 4 - Source data/Figure 4 - Source data 4.tiff]

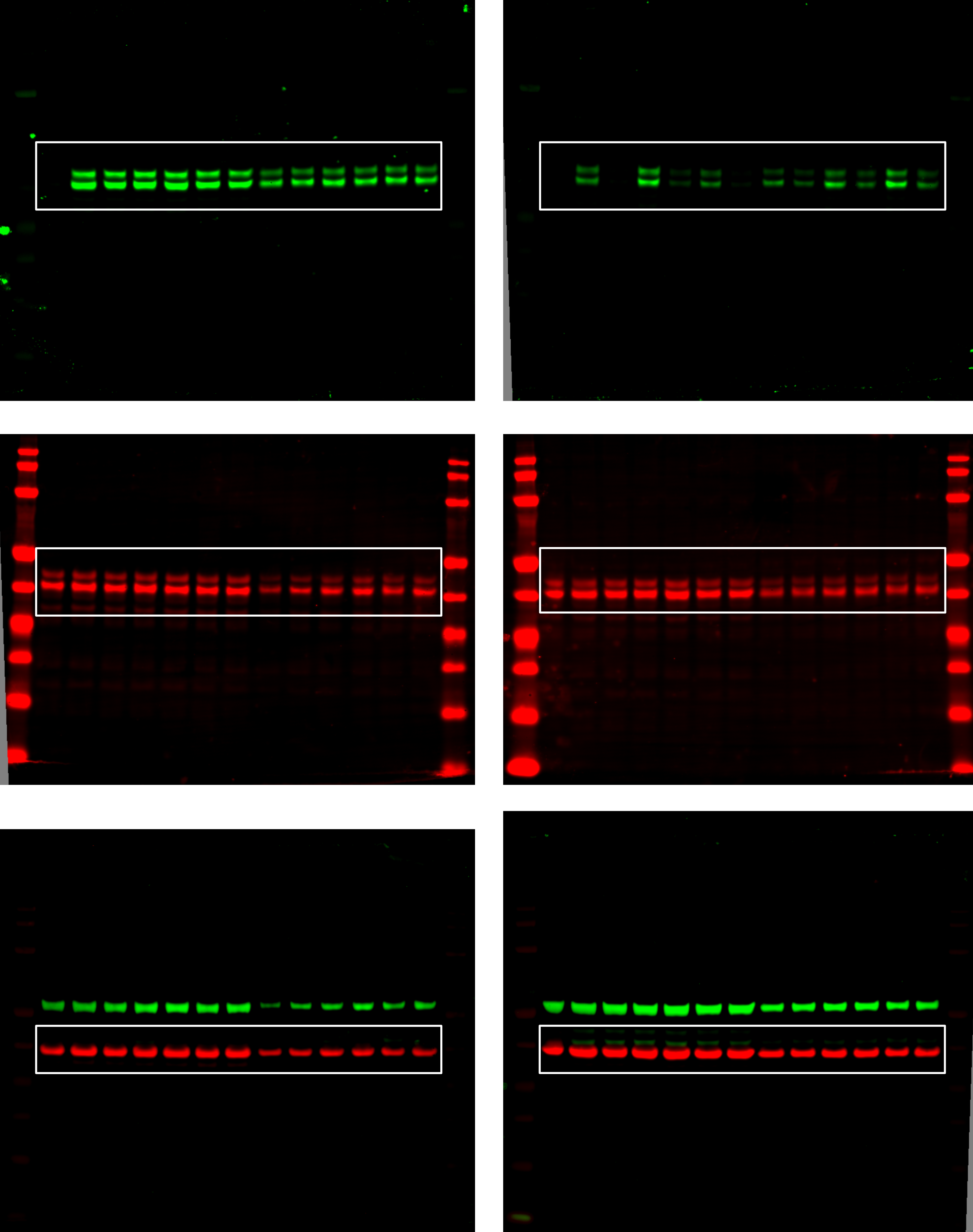

Supplement: Source data 1. [file elife-75545-data1.zip › 2022-02-25 source data 3/Figure 8 - Source data/Figure 8 - Source data 4.tiff]

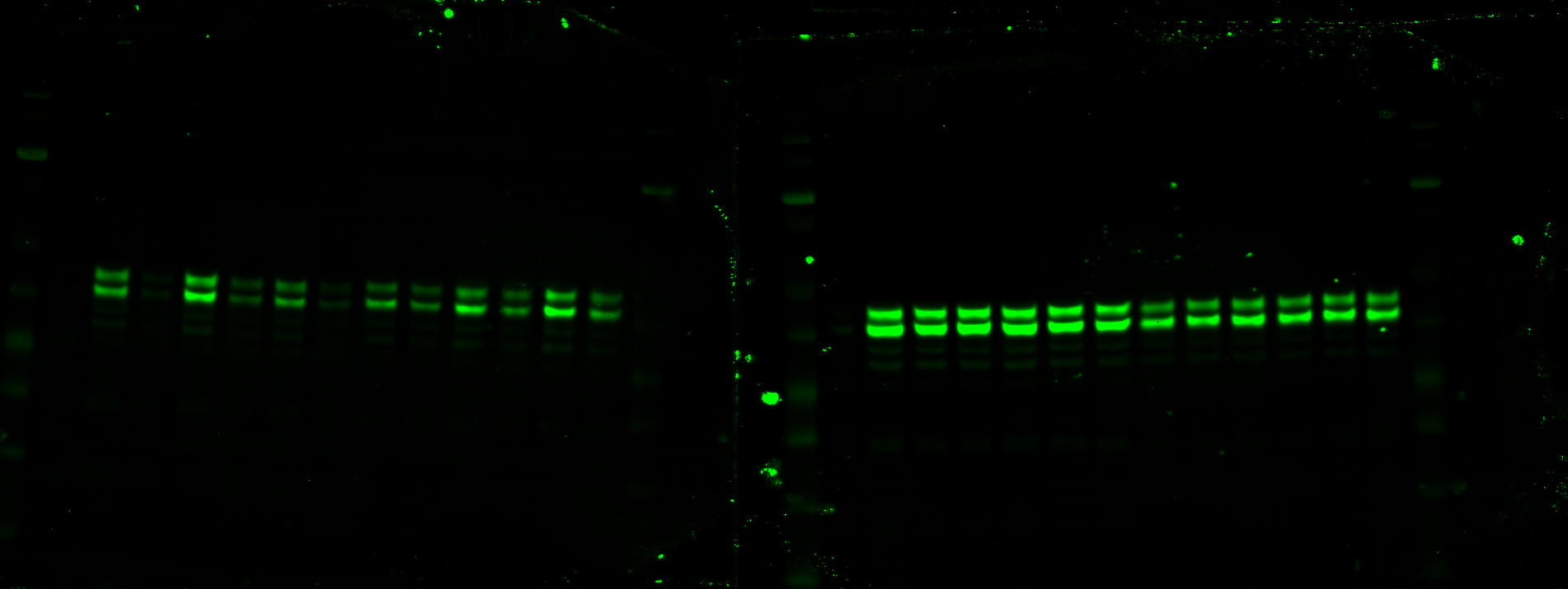

Supplement: Source data 1. [file elife-75545-data1.zip › 2022-02-25 source data 3/Figure 8 - Source data/Figure 8 - Source data 1.tif]

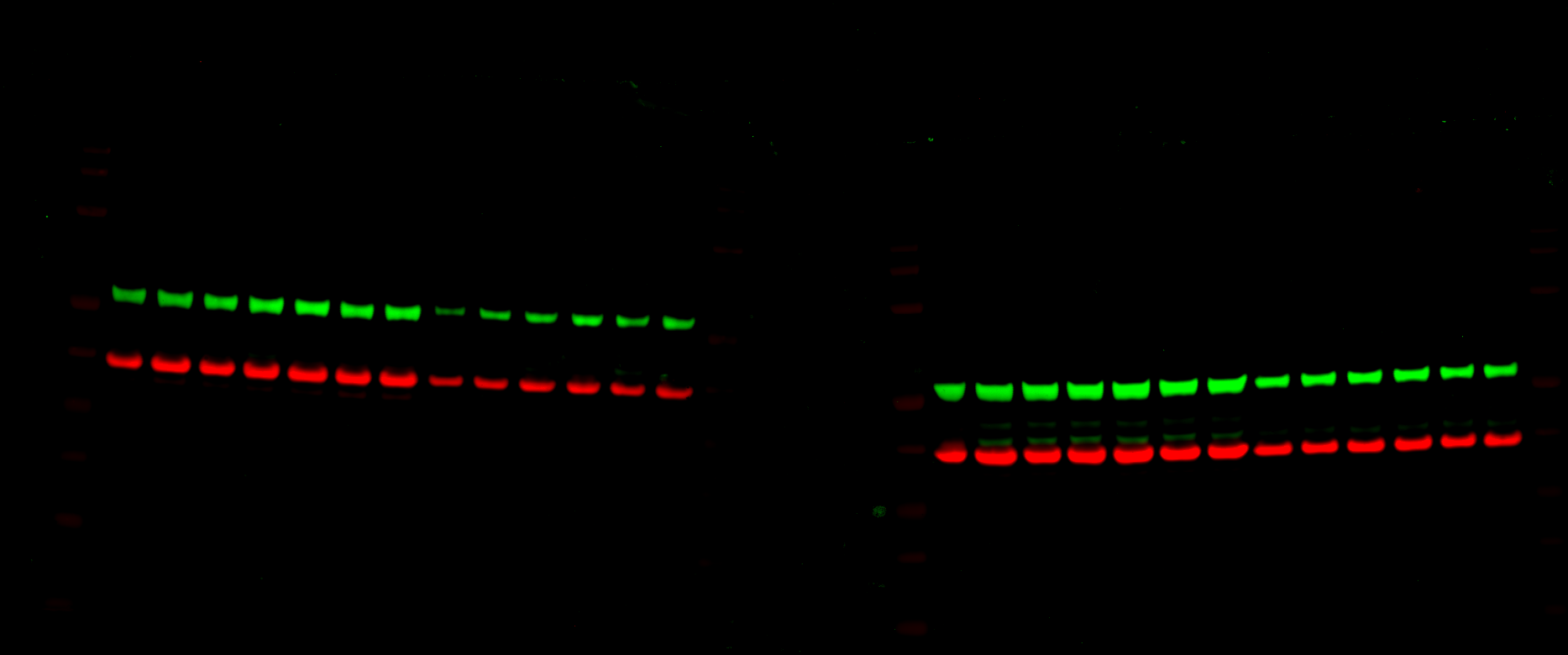

Supplement: Source data 1. [file elife-75545-data1.zip › 2022-02-25 source data 3/Figure 8 - Source data/Figure 8 - Source data 3.tif]

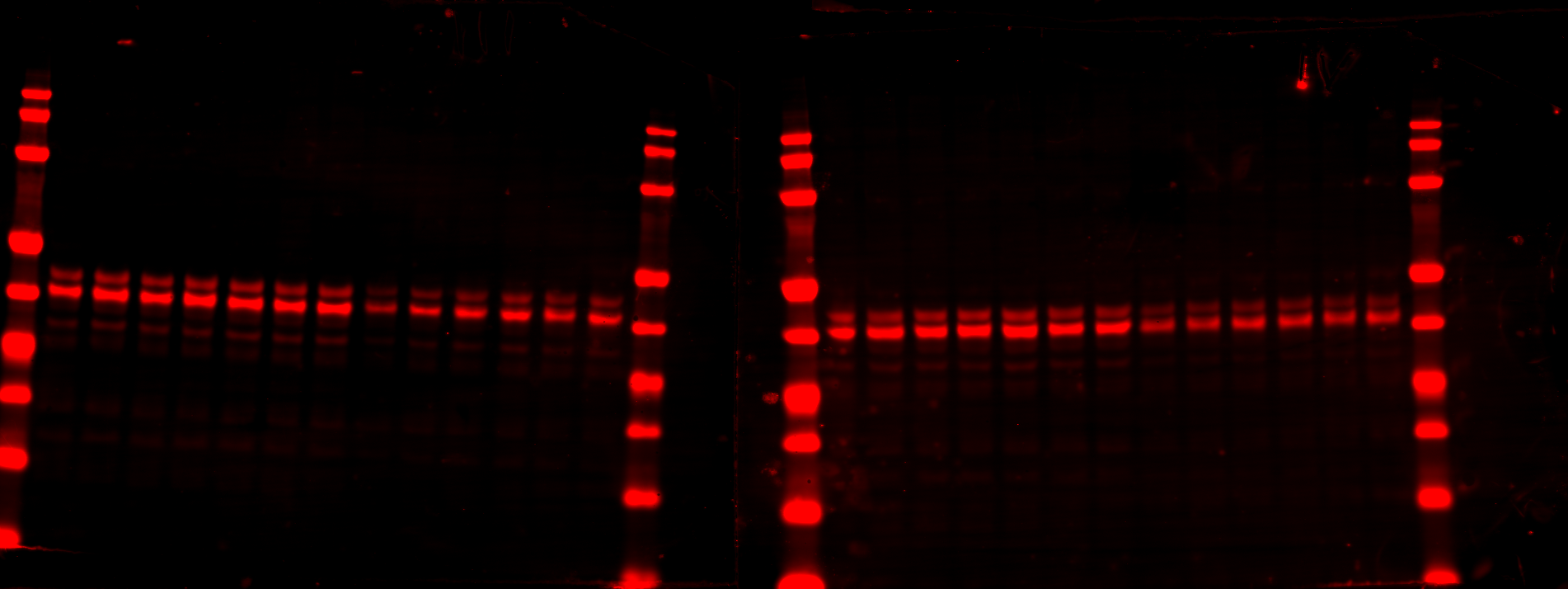

Supplement: Source data 1. [file elife-75545-data1.zip › 2022-02-25 source data 3/Figure 8 - Source data/Figure 8 - Source data 2.tif]

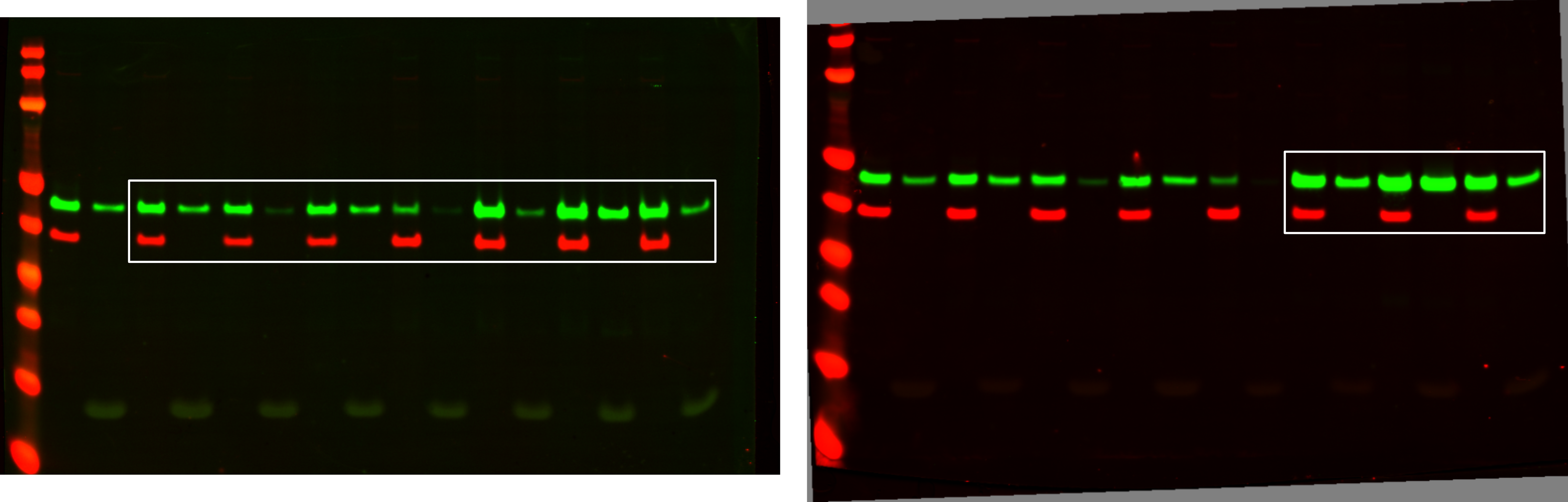

Supplement: Source data 1. [file elife-75545-data1.zip › 2022-02-25 source data 3/Figure 2 - Source data/Figure 2 - Source data 3.tiff]

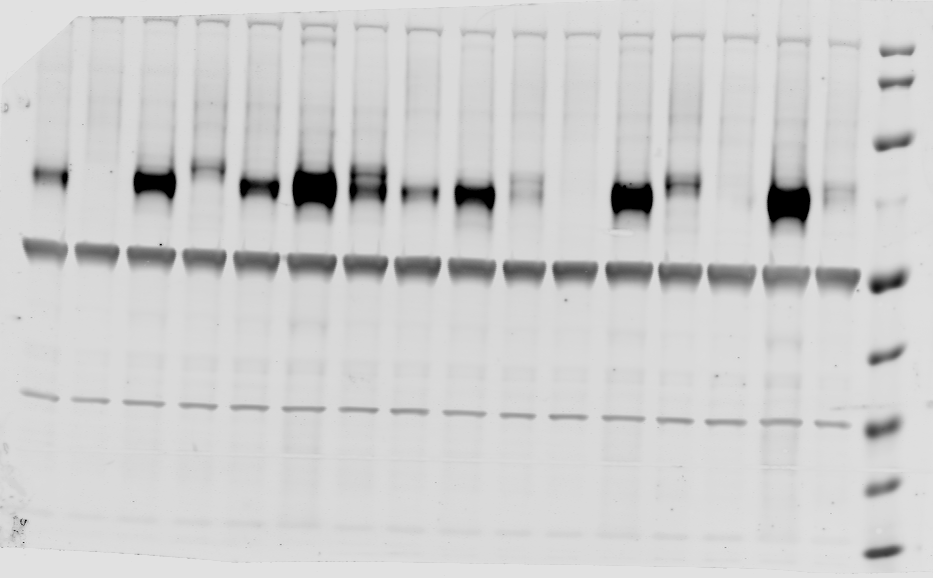

Supplement: Source data 1. [file elife-75545-data1.zip › 2022-02-25 source data 3/Figure 2 - Source data/Figure 2 - Figure Supplement 1 - Source data 1.tif]

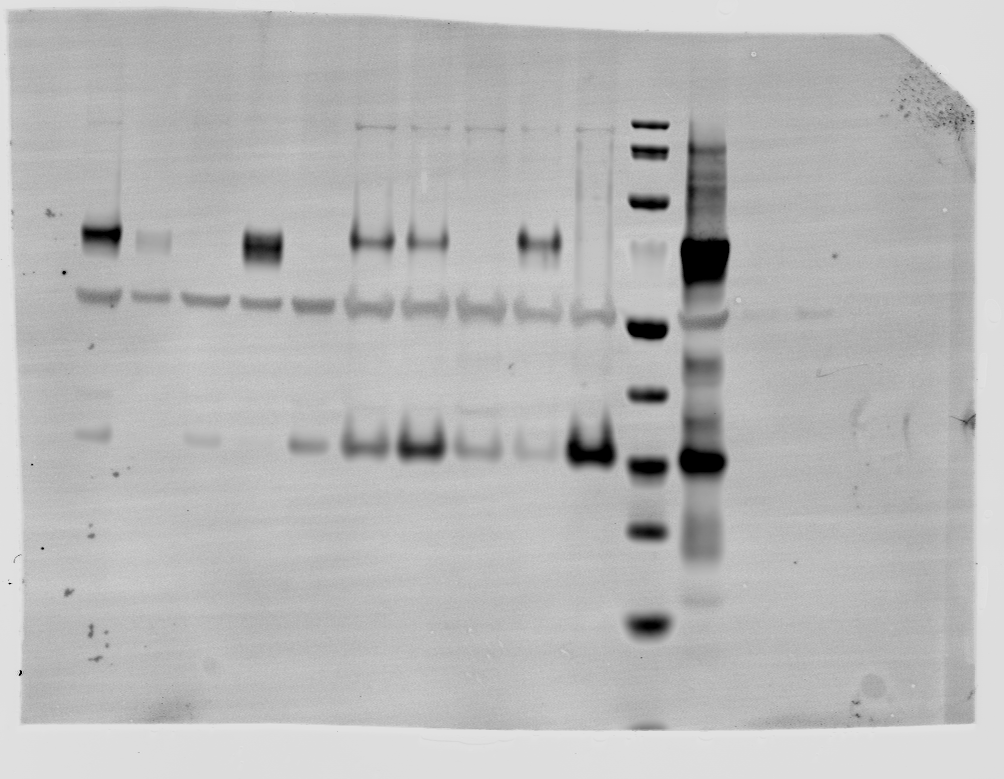

Supplement: Source data 1. [file elife-75545-data1.zip › 2022-02-25 source data 3/Figure 2 - Source data/Figure 2 - Figure Supplement 1 - Source data 2.tif]

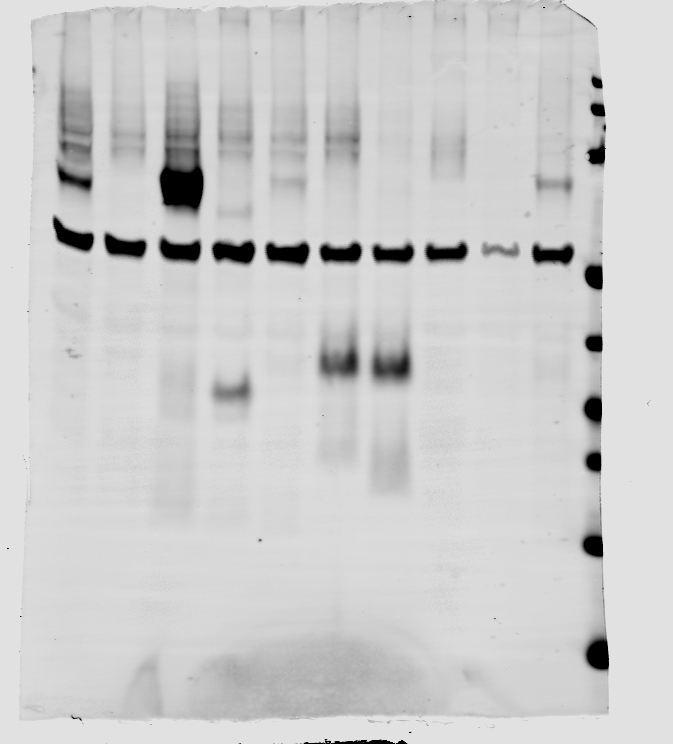

Supplement: Source data 1. [file elife-75545-data1.zip › 2022-02-25 source data 3/Figure 2 - Source data/Figure 2 - Figure Supplement 1 - Source data 3.tif]

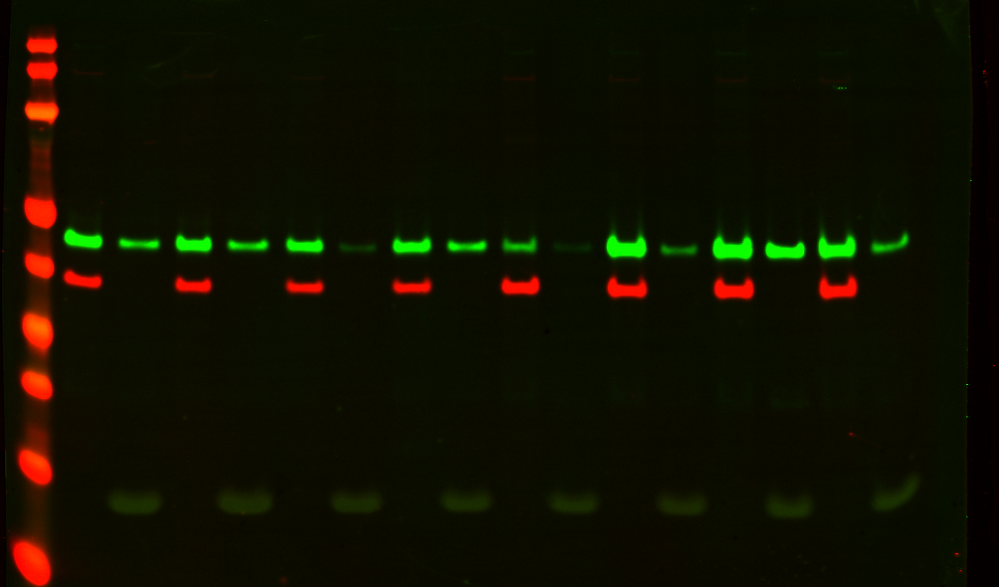

Supplement: Source data 1. [file elife-75545-data1.zip › 2022-02-25 source data 3/Figure 2 - Source data/Figure 2 - Source data 1.png]

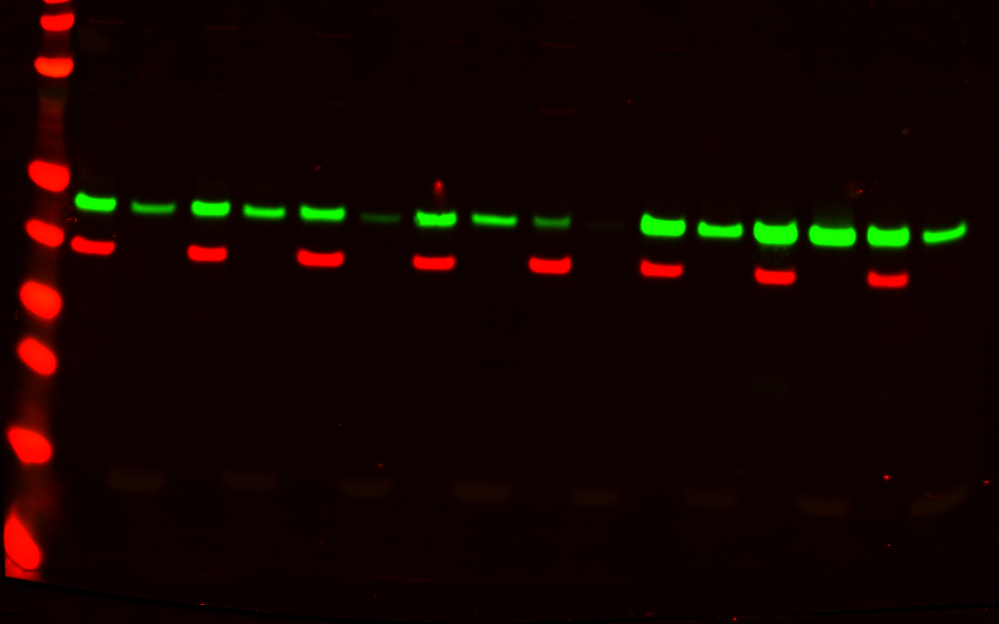

Supplement: Source data 1. [file elife-75545-data1.zip › 2022-02-25 source data 3/Figure 2 - Source data/Figure 2 - Source data 2.png]

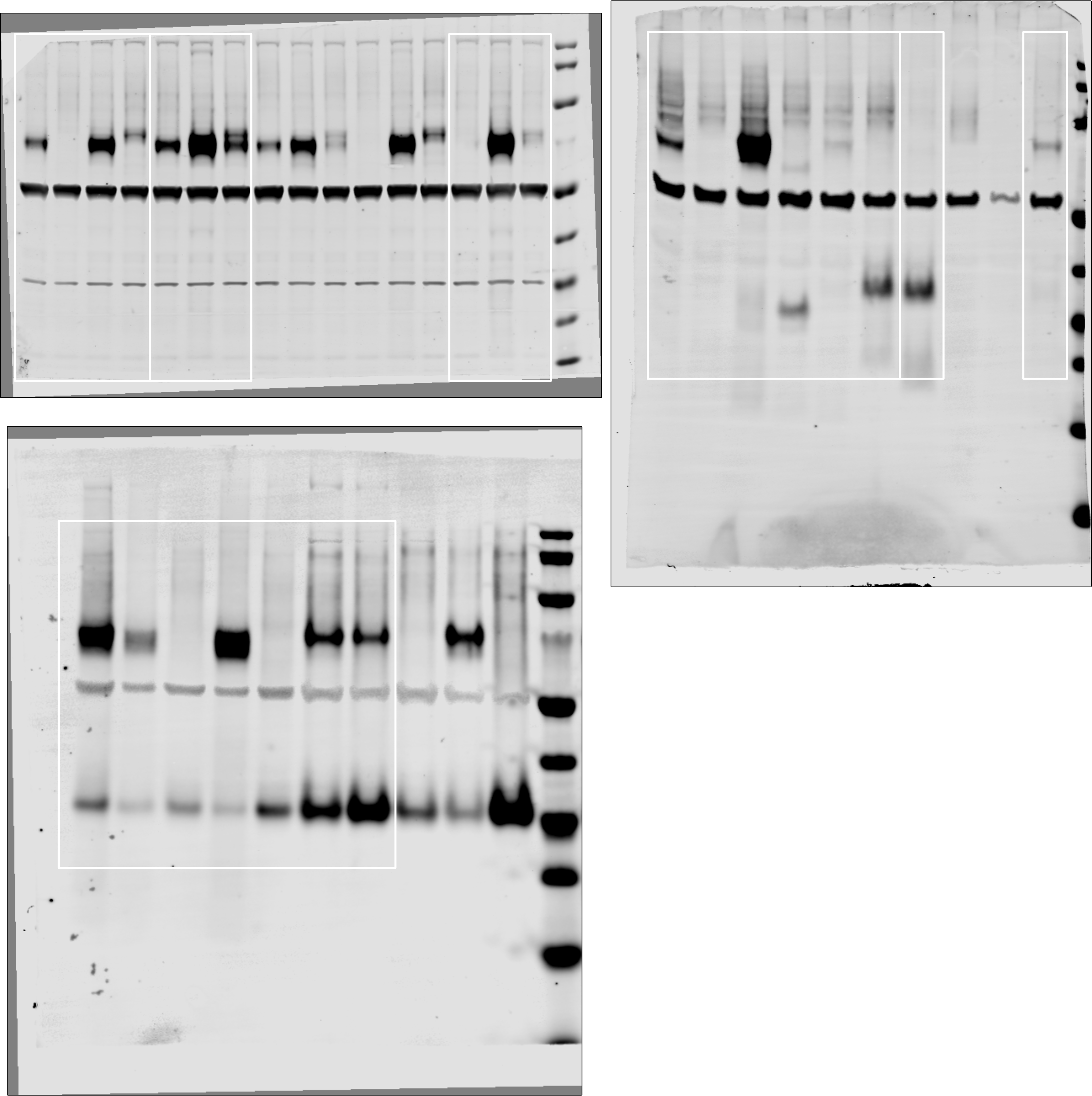

Supplement: Source data 1. [file elife-75545-data1.zip › 2022-02-25 source data 3/Figure 2 - Source data/Figure 2 - Figure Supplement 1 - Source data 4.tiff]

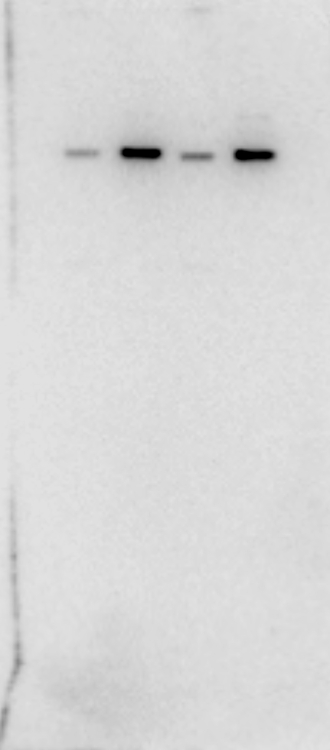

Supplement: Source data 1. [file elife-75545-data1.zip › 2022-02-25 source data 3/Figure 1 - Source data/Figure 1 - Source data 8.jpg]

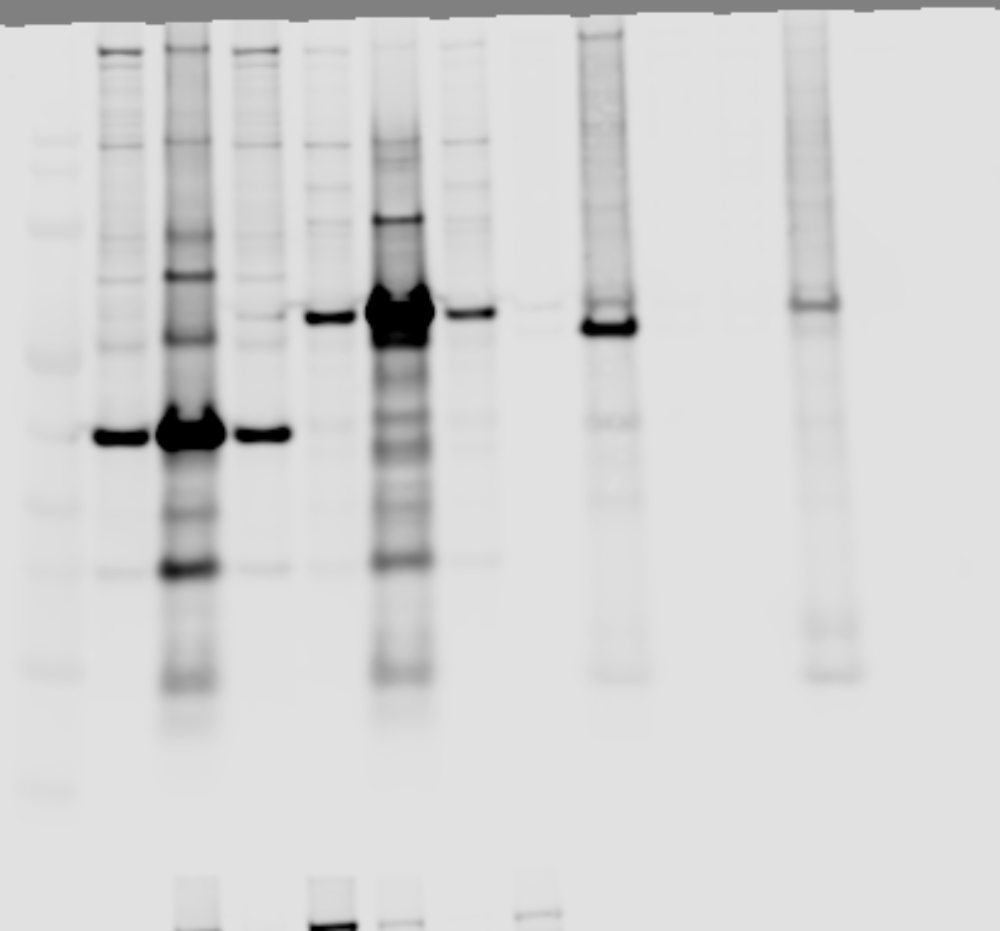

Supplement: Source data 1. [file elife-75545-data1.zip › 2022-02-25 source data 3/Figure 1 - Source data/Figure 1 - Source data 9.jpg]

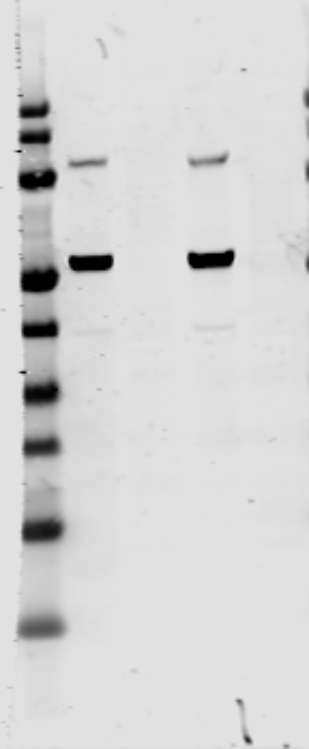

Supplement: Source data 1. [file elife-75545-data1.zip › 2022-02-25 source data 3/Figure 1 - Source data/Figure 1 - Source data 6 .jpg]

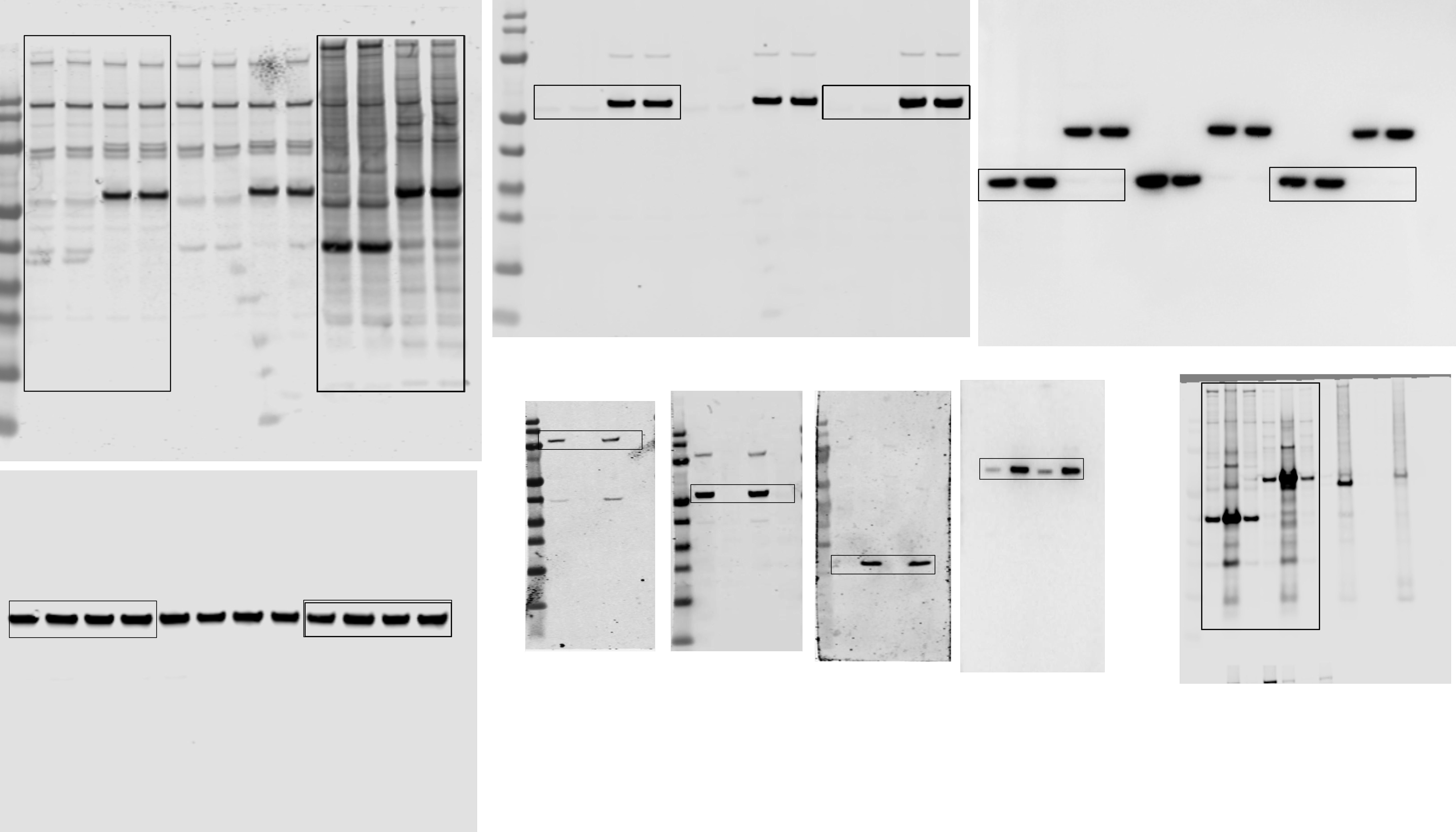

Supplement: Source data 1. [file elife-75545-data1.zip › 2022-02-25 source data 3/Figure 1 - Source data/Figure 1 - Source data 10.tiff]

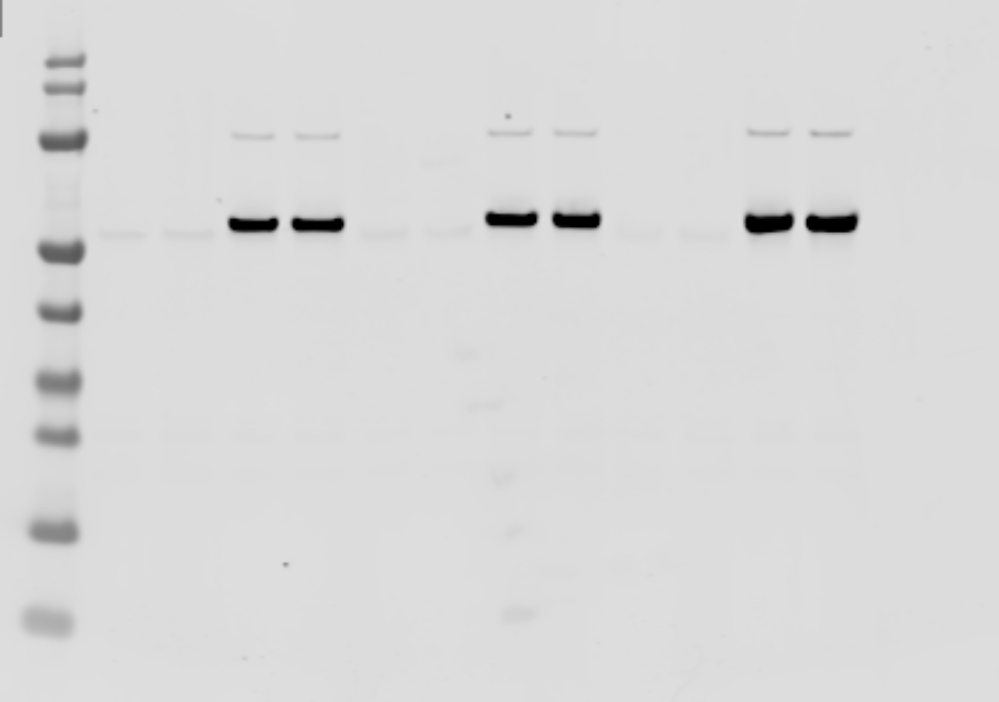

Supplement: Source data 1. [file elife-75545-data1.zip › 2022-02-25 source data 3/Figure 1 - Source data/Figure 1 - Source data 2.jpg]

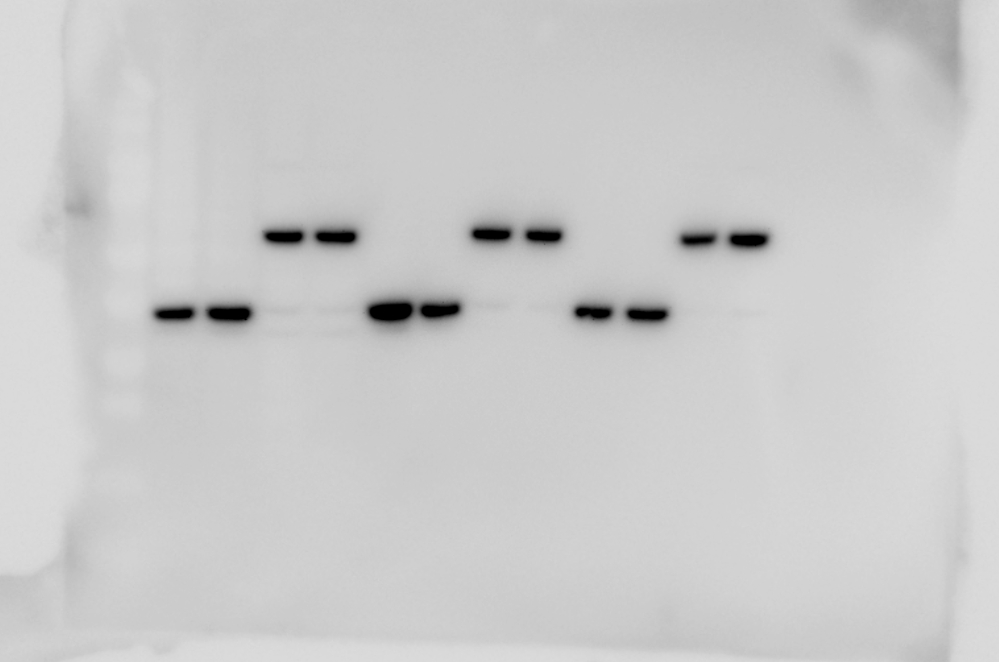

Supplement: Source data 1. [file elife-75545-data1.zip › 2022-02-25 source data 3/Figure 1 - Source data/Figure 1 - Source data 3.jpg]

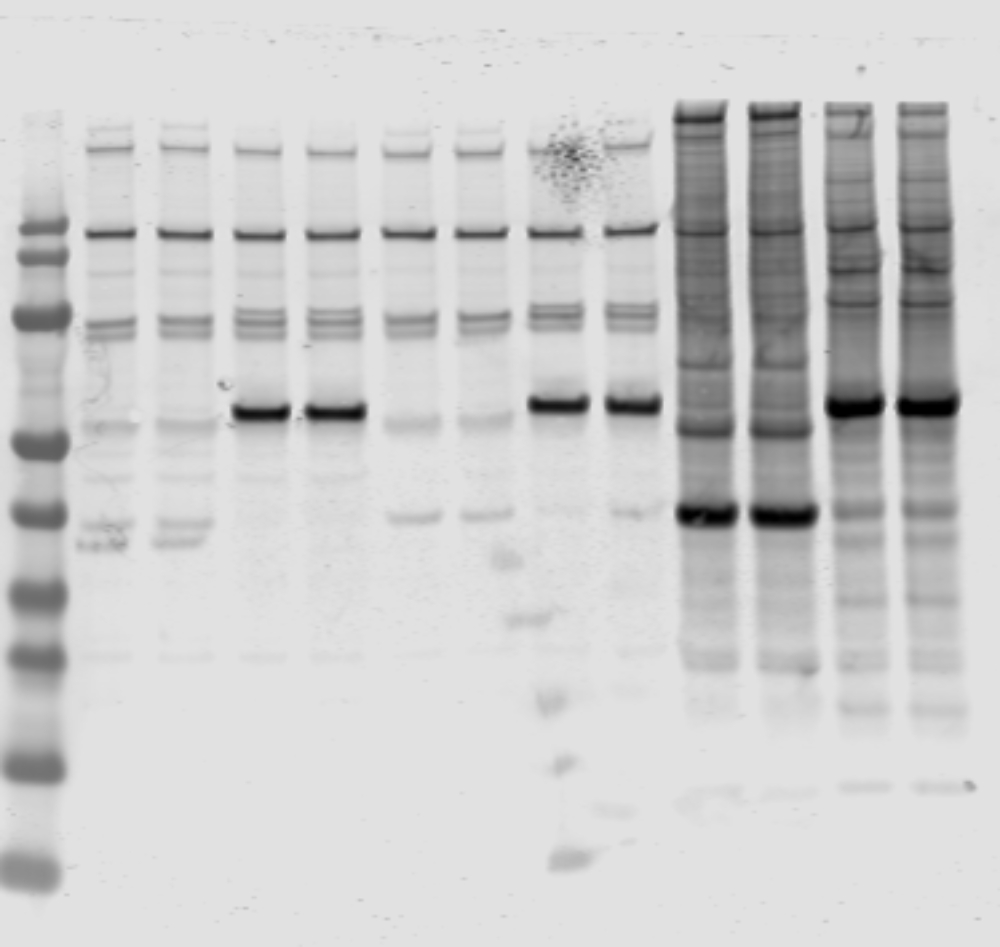

Supplement: Source data 1. [file elife-75545-data1.zip › 2022-02-25 source data 3/Figure 1 - Source data/Figure 1 - Source data 1.jpg]

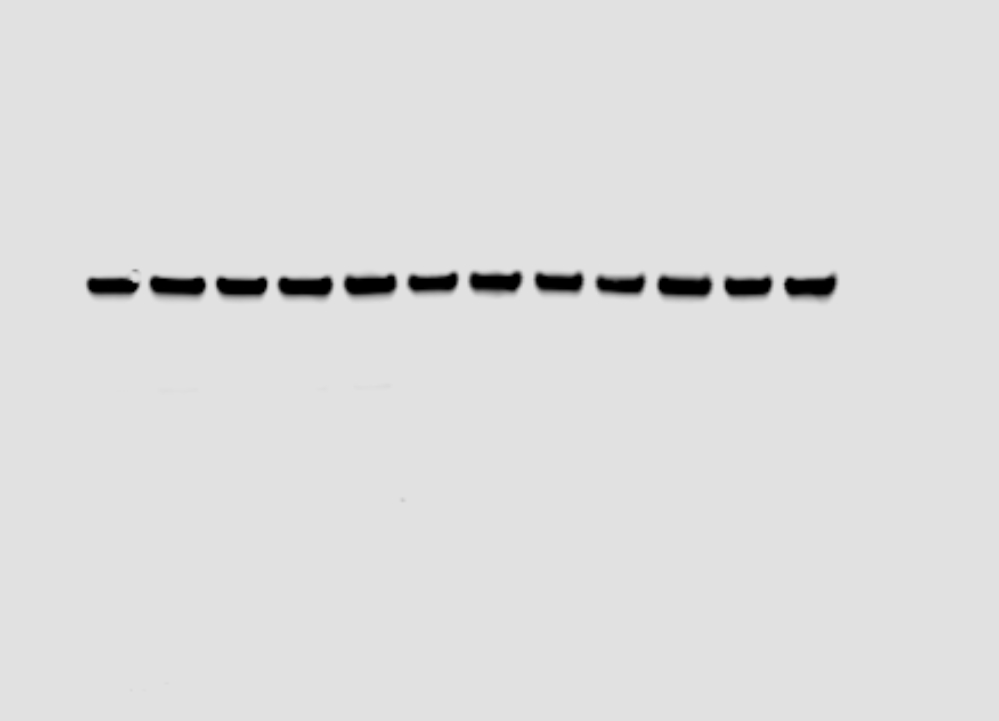

Supplement: Source data 1. [file elife-75545-data1.zip › 2022-02-25 source data 3/Figure 1 - Source data/Figure 1 - Source data 4.jpg]

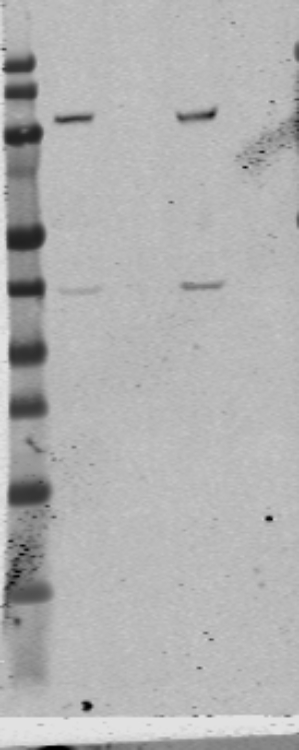

Supplement: Source data 1. [file elife-75545-data1.zip › 2022-02-25 source data 3/Figure 1 - Source data/Figure 1 - Source data 5.jpg]

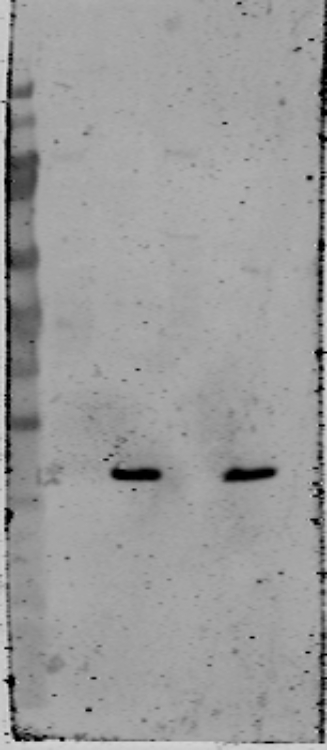

Supplement: Source data 1. [file elife-75545-data1.zip › 2022-02-25 source data 3/Figure 1 - Source data/Figure 1 - Source data 7.jpg]
